# Undernutrition and Associated Factors Among Children Aged 6–23 Months in Dessie Town, Northeastern Ethiopia, 2021: A Community Based Cross-Sectional Study

**DOI:** 10.3389/fped.2022.916726

**Published:** 2022-07-08

**Authors:** Tesfamaryam Sewenet, Mulugeta W/Selassie, Yosef Zenebe, Wondwossen Yimam, Lebasie Woretaw

**Affiliations:** ^1^Department of Public Health, Tossa Medical and Surgical Specialty Centre, Dessie, Ethiopia; ^2^Department of Pediatrics and Child Health Nursing, Wollo University, Dessie, Ethiopia; ^3^Department of Psychiatry, Wollo University, Dessie, Ethiopia; ^4^Department of Comprehensive Nursing, Wollo University, Dessie, Ethiopia; ^5^Department of Environmental Health, Wollo University, Dessie, Ethiopia

**Keywords:** undernutrition, associated factors, children, Dessie, Ethiopia

## Abstract

**Background:**

Globally about 159, 101, and 52 million children are stunted, underweight, and wasted, respectively. According to the 2016 Ethiopian Demographic and Health Survey, about 38% of Ethiopian children are stunted and 46, 28.4, and 9.8% of children in Amhara Region are stunted, underweight and wasted, respectively. This study aimed to assess undernutrition and associated factors among children aged 6-23 months old at Dessie town, 2021.

**Method:**

A community-based cross-sectional study was conducted from October – November 2021 in Dessie Town. A total of 421 Mothers/caregivers with children aged 6-23 months old were selected by a systematic sampling method from the health extension registration book. Epi-data 3.01 was used for data entry, SPSS version 20 for statistical analysis, and WHO Anthro version 3.2.2 software for calculating the *z* scores. Binary logistic regression and multivariate logistic regression were used to analyze the data. AOR with 95% CI and *P*-values less than 0.05 were considered to see the statistical significance.

**Results:**

A total of 421 mothers or care givers paired with 6-23 months old children participated in the study. The prevalence of stunting, underweight, wasting were 36.8% (95% CI: 32%, 41.6%), 27.6% (95% CI: 23.6%, 32.2%), and 11.5% (95% CI: 8.4%, 14.7%) respectively. Sex of the child (AOR = 1.55; 95% CI: 1.02, 2.34), handwashing practice (AOR = 2.32; 95% CI: 1.05, 5.11) and maternal family planning use (AOR = 0.39; 95% CI: 0.19, 0.77) were significantly associated with stunting. Age of child 12-17 months (AOR = 4.62; 95% CI: 2.65, 8.06) and sex of the child (AOR = 1.93; 95% CI: 1.21, 3.07) were associated with underweight. Age of child 12-17 months (AOR = 2.25; 95% CI: 1.06, 4.78) and treatment of drinking water (AOR = 0.21; 95% CI: 0.07, 0.59) were associated with wasting.

**Conclusion and Recommendation:**

In this study, the prevalence of undernutrition among children aged 6-23 months was higher for stunting (36.8%), underweight (27.6%) and wasting (11.5%) compared to WHO classification. Improved access to water, hygiene and sanitation, family planning services, avoiding gender discrimination during child feeding, and age-appropriate feeding practices are recommended. Moreover, implementation of public policies on food and nutrition is required for children 6-23 months of age.

## Introduction

The nutritional status of children is important because it determines their health, growth, development, academic performance, and progress in life. All children have the right to adequate nutrition, which is essential for their health (1). Good nutrition has been reported to be the cornerstone for survival, health, and development in current and future generations (2). Malnutrition generally refers to both undernutrition, including stunting, wasting, and underweight, and over nutrition, including overweight, obesity, and diet-related non-communicable diseases (such as heart disease, stroke, diabetes, and cancer) (3).

Stunting (inadequate length for age) captures early chronic exposure to undernutrition; wasting (inadequate weight for height) captures acute undernutrition; and underweight (inadequate weight for age) is a composite indicator that includes elements of stunting and wasting, and poor nutrition in the first 1,000 days of life can have irreversible consequences. For millions of children, it means that they are, forever, stunted and nutritional status is influenced by three broad factors: food, health, and care. Optimal nutritional status results when children have access to affordable, diverse, nutrient-rich food; appropriate maternal and child-care practices; adequate health services; and a healthy environment including safe water, sanitation, and good hygiene practices. These factors directly influence nutrient intake and the presence of disease. The interaction between undernutrition and infection creates a potentially lethal cycle of worsening illness and deteriorating nutritional status (4).

Insufficient dietary diversity is a problem at any age but is particularly critical for children between the ages of 6-23 months during the complementary feeding period, who need food containing essential nutrients for normal physical and mental development. Those who eat four or more foods from the seven food groups daily have the minimum recommended dietary diversity. Under the assumption that they consume at least one animal source food and at least one fruit or vegetable in addition to staple foods (5).

Lack of diversity in dietary intake is a serious problem among young children and women of reproductive age in developing countries. Dietary diversity is highly connected with height for age *Z* score and growth among young children (6). Therefore, this study aimed to assess undernutrition and associated factors among 6- to 23-month-old children in Dessie town.

## Materials and Methods

### Study Area and Period

This study was conducted in the Dessie Town administration. There are two governmental and three private hospitals in Dessie town. Based on the 2007 national census conducted by the Central Statistical Agency of Ethiopia (CSA), Dessie town has a total population of 151,174, of whom 72,932 are men and 78,242 are women. A total of 2,924 children 6-23 months of age live in Dessie Town, and 770 children 6-23 months of age live in the selected three sub cities. The study was conducted from October – November 2021.

### Study Design

Community-based cross-sectional study design was conducted.

### Source Population

The source population the source population was all mothers/caregivers with a child aged 6-23 months who resided in Dessie Town.

### Study Population

The study population was all children aged 6-23 months who resided in the selected sub cities during the study period.

### Inclusion and Exclusion Criteria

In this study, all children aged 6-23 months living in the selected sub cities were included, and mothers/caregivers with communication barriers or mental problems and children with physical deformities were excluded.

### Sample Size Determination

The sample size was calculated using Epi Info version 7.2 statistical software and by considering the following assumption. Based on findings from the EDHS 2016 report, the prevalence of stunting, wasting, and underweight in Amhara Regions was 46, 9.8, and 28.4%, respectively (7). Therefore, the total sample sizes were calculated with a margin of error of 0.05, 95% confidence level, and 10% non-response. Finally, 421 children 6-23 months of age were taken from the study area.

### Sampling Technique and Procedure

A multistage sampling method was used for this study. The sampled sub city was selected by using the lottery method from the 10 sub cities, and lists of under-five children were obtained from the sub city responsible person (Health extension worker housing registration). The sampling technique was a systematic random sampling technique. The total samples were proportionally allocated for the three sub cities, and the allocated sample size was selected by a systematic random sampling technique and determination of the *K* value, *K* = 770/421 = 1.8 (approximately = 2). The first subject was selected by the lottery method, which was number 1.

### Data Collection Procedures

Data were collected through interviewer-administered structured questionnaires and anthropometric tools. The data were collected by three diploma health extension workers working in the respective kebele (a total of three supervisors and nine data collectors were recruited from the three kebeles), and target children were selected using systematic random sampling from the document. This document was used to find their home/residence address. Finally, after obtaining the target children by using a structured questionnaire, caregivers/mothers were interviewed, and the length and weight of the child were measured. The date of birth was obtained from EPI cards and cross-check with neighbors. Children wore similar clothes and without shoes while measuring weight. During length measurement, children were in a recumbent position and Frankfurt plane 90 degrees in length. To measure weight, a medically acceptable weight scale (Digital SECA, made in Germany, model 874 1021659, serial number 5874269114011, graduation of 0.1 kg and measuring up to 150 kg) was used.

### Dependent Variables

Stunting (Yes/No), Wasting (Yes/No) and Underweight (Yes/No).

### Independent Variables

Socioeconomic and demographic factors (age, sex, income, educational status (mother and father), maternal and child health status (diarrhea, pneumonia, TB &HIV immunization status, F/P, ANC, PNC, others), child feeding practice (initiation of complementary feeding, breastfeeding, minimum dietary diversity, and household food security status) and WASH factors (water availability, sanitation, handwashing) were the independent variables.

### Operational Definitions

Dietary diversity: The number of food or food group children from 6-23 months of age consumed for 24 h in the study period/reference period (from October to November 2021).

High dietary diversity: Children are 6-23 months of age who receive four or more food groups of the seven food groups (8).

Low dietary diversity: Children are 6-23 months of age who receive fewer than four food groups of the seven food groups (8).

Minimum dietary diversity: Children 6–23 months of age who receive foods from 4 or more food groups from the seven during the study period (from October to November 2021) (8).

Caregiver: A person who provides direct care for children (parents or other caregivers).

Stunting: Height -for- age <−2 standard deviations (SDs) from the median of the WHO reference population (9).

Wasting: Weight-for-height <−2 SD from the median of the WHO reference population (9).

Underweight: Weight-for-age <−2 SD from the median of the WHO reference population (9).

Food security: Assessed by nine food insecurity questions that hold the occurrence of food insecurity in the previous month (0 – 3 = secure, >3 = insecure) (10).

Kebele: A “Kebele” is a small administrative unit that comprises up to 5,000 households (11).

### Data Collection Tool and Procedures

Food and Nutrition Technical Assistant (FANTA) tool for dietary diversity questions based on 24 recall periods from mother or child caregivers who are responsible for food preparation. The questionnaires have a minimum score of 0-7 (0-3 = low Dietary Diversity Score (DDS), 4-5 = medium DDS, 6-7 = high DDS). Though, Food and Nutrition Technical Assistant (FANTA) tool for dietary diversity classified in to three categories, previous studies conducted here in Ethiopia used the high and low dietary diversity classifications. It is because the medium category was not applicable for intervention here in our country, Ethiopia. Based on this reason, we all the authors modified this tool in to two categories as high (>4 food groups) and low dietary diversity (<4 food groups) (Reference no. 8). The Household Food Insecurity Access Scale (HFIAS) was used to assess the household food security status of households.

Food security status was categorized into food security if the score was 0-3 and insecure if the score was greater than three. A weight scale (digital SECA, made in Germany, model 8741021659, serial number 5874269114011 and Graduation of 0.1 kg and measuring up to 150 kg and capable of reading to the nearest 0.1 kg) was used to measure the weight of the child. A horizontal wooden length board was used to measure the length in a recumbent position, which was read to the nearest 0.1 cm. Weight and length measurements were made three times, and then the average was computed. All completed questionnaires and weight measuring instruments were checked and calibrated daily (calibration was performed before weighing every child by setting it to zero and checking by putting a 1 kg iron rod before taking children’s weight) and regularly supervised on a daily basis by trained supervisors, and each questionnaire was checked for its completeness, accuracy, and consistency by the primary investigator.

### Data Quality Assurance

To ensure data quality, the recruited data collectors and supervisors were trained for two successive days. The questionnaire was developed in English and translated and adopted into locally acceptable “Amharic” and translated back to English to ensure consistency in the asking of questions by the interviewers. A pre-test was carried out on 10% of the actual sample size in another kebele to determine the acceptability of the question to be asked, appropriateness of the methods, reaction, and willingness of the respondents.

### Data Processing and Analysis

To analyze the data, the dietary diversity score was determined by asking the food groups they ate within 24-h periods during the data collection period. Each food group has one mark, and the total food groups were scored from seven. Data processing and analysis were employed by the appropriate software Epi-data version 3.01 software for data entry. After the completion of data entry, recorded data were exported to SPSS version 20 for data analysis, and nutrition-related data (sex, age, height, and weight) were transferred to WHO Anthro version 3.2.2 to determine stunting, wasting, and underweight. All the findings are described in detail and summarized in percentages, mean + = SD, tables, and graphs, and for each outcome variable, binary logistic regression was performed. Binary logistic regression was used to indicate the gross association between each independent variable and the outcome variable. Then, those candidate variables that were filtered from the binary logistic regression (*P* value < 0.2) were moved to multivariable logistic regression, and adjusted odds ratios with 95% confidence intervals were reported, so the level of statistical significance was considered at a *p* value of <0.05 from the final model.

### Ethical Approval and Consent to Participate

Ethical clearance was obtained from Wollo University College of Medicine and Health Sciences ethical review committee and a written letter was given to the selected sub-cities. All the study participants were informed about the purpose of the study and assured confidentiality of the responses. Written consent was obtained from each participant. There are no known risks to a participant who takes part in this study. Accountability, confidentiality, neutrality, and academic honesty were maintained throughout the study.

## Results

### Sociodemographic Characteristics of Participants

A total of 421 children with their mothers or caregivers were included in the study, giving a response rate of 100%. Out of these households, 400 (95%) were mothers, and the remaining 21 (5%) were caregivers. The majority of mothers/caregivers were found in the age range of 25-29 years 181 (43%). The mean age of the respondents was 28.8 (± 4.76), and the mean ± SD age of the respondents at first birth was 22.2 (± 3.36) years. The majority of mothers were married (365, 86.7%) (Table 1).

**TABLE 1:** Sociodemographic characteristics of the study participants, Dessie Town, South Wollo Zone, Northeastern Ethiopia, 2021.

Variable		Frequency	%
Maternal age (in a year)	15-19 20-24 25-29 30-34 >35	6 59 181 100 75	1.4 14 43 23.8 17.8
Relation with the child	Mother Caregiver	400 21	95 5
Maternal age at first birth	<20 20-34	212 209	50.4 49.6
Household head	Husband Wife Grandmother/father	233 184 4	55.3 43.7 1
Marital status	Single Married Separated Widow	31 365 20 5	7.4 86.6 4.8 1.2
Ethnicity	Amhara Oromo Tigray	393 19 9	93.3 4.5 2.1
Religion	Orthodox Muslim Protestant	253 110 58	60.1 26.1 23.8
Maternal educational level	Without formal education Elementary (1-8) Secondary & above	33 173 215	7.8 41.1 51.1
Paternal educational level	Without formal education Literate (read & write) Elementary (1-8) Secondary & above	13 3 136 269	3.0 0.7 32.3 63.9
Maternal occupation	Civil servant Self- employer Daily worker Housewife	118 52 38 213	28 12.4 8.9 50.6
Paternal occupation	Civil servant Self- employer Daily worker	216 143 62	51.3 34 14.7
Monthly income	<1,000 ETB (less than $ 21) 1001-2000 ETB ($ 21 - $ 42) >2001ETB (greater than $ 42)	31 170 220	7.4 40.4 52.3
Family size	<5 >5	415 6	98.6 1.4
Participate in decision making on the use of money	No Yes	168 253	39.9 60.1

### Child Feeding Practice and Health Care Characteristics

From a total of 421 study participants, approximately 227 (53.9%) were male, and 194 (46.1%) were female. 66 (15.7%) between 18 and 23 months. The mean age of the children was 12.56 (± 4.42) months. Three hundred eighty-six (91.7%) children were not taking prelacteal food. Of the 35 children, 27 (77.1%) took milk (Table 2).

**TABLE 2:** Child feeding practices and health care characteristics, Dessie Town, South Wollo Zone, Northeastern Ethiopia, 2021.

Variable	Category	Frequency	%
Child sex	Male Female	227 194	53.9 46.1
Child age (in months)	6-8 9-11 12-17	89 101 231	21.1 24 54.2
Child ever breastfeed	No Yes	168 253	39.9 60.1
Duration of breastfeeding	<12 months 12-24 months	368 53	87.4 12.6
Time of initiation of breast milk	Not known (not specified) within 1 h 1-24 h after24 h	3 240 158 20	7 5 37.5 4.8
Prelacteal feeding	No Yes	386 35	91.7 8.3
Type of prelacteal food	Milk Butter Sugar	27.3 5	77.1 8.6 14.3
Colostrum given	No Yes	24 397	5.7 94.3
Start complementary food (at 6 months)	No Yes	9 412	2.1 97.9
Time of initiation of complementary food	Before 6 months At 6 months After 6 months	325 87 9	77.2 20.6 2.2
Frequency of complementary food	<3 times >3 times	101 320	24 76
Method of feeding	Spoon Cup Hand Maternal support Bottle feeding	122 78 46 2 173	29 18. 10.9 0.5 41.1
Knowledge on IYCF	No Yes	22 399	5.2 94.8
Source of IYCF knowledge	No Health professional Friends Media Family	22 308 22 37 32	5.2 73.2 5.2 8.8 7.6

The mean dietary diversity score was 2.06 (± 0.75). In this study, 105 (24.9%) had low dietary diversity scores (<4 food groups), 316 (75.1%) had high dietary diversity scores (>4 food groups) (Table 3).

**TABLE 3:** Dietary diversity habits of children 6–23 months, Dessie Town, South Wollo Zone, Northeastern Ethiopia, 2021.

Variable	Category	Frequency	%
Grain, roots, tubers	No Yes	63 358	15 85
Vit-A rich plant foods	No Yes	134 287	31.8 68.2
Other fruit or vegetables	No Yes	196 225	46.6 53.4
Meat, poultry, fish & seafood	No Yes	263 158	62.5 37.5
Egg	No Yes	277 144	65.8 34.2
Pulse, legumes & nuts	No Yes	197 224	46.8 53.2
Milk and milk product	No Yes	191 230	45.4 54.6

#### Maternal and Child Health Characteristics

Of the 421 participants, 367 (87.2%) mothers attended family planning services, and 14 (3.3%) mothers reported HIV in their blood. Approximately 347 (82.4%) of mothers practiced eating extra food during pregnancy and lactation, 292 (69.4%) of children took vitamin A supplementation, 170 (40.4%) of children had diarrheal disease in the past 6 weeks, 3 (0.7%) of them tested positive for HIV, and 302 (71.7%) of children completed immunization (Table 4).

**TABLE 4:** Maternal and child health characteristics, Dessie Town, South Wollo Zone, Northeastern Ethiopia, 2021.

Variable	Category	Frequency	%
Maternal family planning	No Yes	54 367	12.8 87.2
ANC	No Yes	12 409	2.9 97.1
PNC	No Yes	22 399	5.2 94.8
Maternal HIV status	No Yes	407 14	96.7 3.3
Extra food during pregnancy & lactation	No Yes	74 347	17.6 82.4
Child Vit-A	No Yes	129 292	30.6 69.4
Diarrheal disease (past 6 weeks)	No Yes	251 170	59.6 40.4
Having fever (past 2 weeks)	No Yes	295 126	70.1 29.9
A child having ARTI (past 2 weeks)	No Yes	351 70	83.4 16.6
Child Having TB	No Yes	415 6	98.6 1.4
Child HIV status	No Yes	418 3	99.3 0.7
Children Infected by measles (past 1years)	No Yes	417 4	99 1
Complete immunization (child)	No Yes	119 302	28.3 71.7

### Household Food Insecurity Assessment

From a total of 421 study participants, 204 (48.5%) were food secure and 217 (51.5%) were food insecure (Table 5).

**TABLE 5:** Household food insecurity access-related conditions, Dessie Town, South Wollo Zone, Northeastern Ethiopia, 2021.

Variable	Frequency	%
Food secured	204	48.5
Food in-secured	217	51.5

#### WASH Factors

Of the 421 households participating in the study, 411 (97.6%) had improved water sources. Approximately 402 (95.5%) used treated water as a source of drinking water, 381 (90.5%) had knowledge on critical times of handwashing, and 320 (76%) were using the improved toilet (Table 6).

**TABLE 6:** Family water and sanitation characteristics, Dessie Town, South Wollo Zone, Northeastern Ethiopia, 2021.

Variable		Frequency	%
Observe caregiver’s hand	Unclean Clean	21 400	5 95
Observe the child’s hand	Unclean Clean	26 395	6.2 93.8
Household water source	Unimproved (tanker truck/cart) Improved	10 411	2.4 97.6
Water container lids	If any did not have lids If all have lids	37 384	8.8 91.2
Trips spent to fetch water	Three or more trips/day Fewer than 3 trips/day	46 375	10.9 89.1
Treatment on drinking water	Not treated Treated	19 402	4.5 95.5
Handwashing at a critical time	Neither soap/ash used If soap/ash was used	43 378	10.2 89.8
Knowledge on critical times of handwashing	Do not list critical times List critical times	40 381	9.5 90.5
Child bath per day	Bathed less than once a day Bathed at least once a day	85 336	20.2 79.8
Latrine used by the family	Unimproved Improved	101 320	24 76

### Nutritional Status of Children Aged 6-23 Months

The analysis of the three indices height-for-age, weight-for-age and weight-for-length (HAZ, WAZ and WHZ) revealed that 36.8% (95% CI (32.%, 41.6%), 27.6% (95% CI (23.6%, 32.2%) and 11.5% (95% CI (8.4%, 14.7%) of the 416 children included in the study were found to be stunted, underweight and wasted, respectively, and 5 (0.01%) were overweight, but since it did not fulfill the assumption of the chi-square test and logistic regression, they were not included in the analysis, and 416 children were ultimately included in the analysis.

### Stunting/Height-For-Age/

**FIGURE 1:**
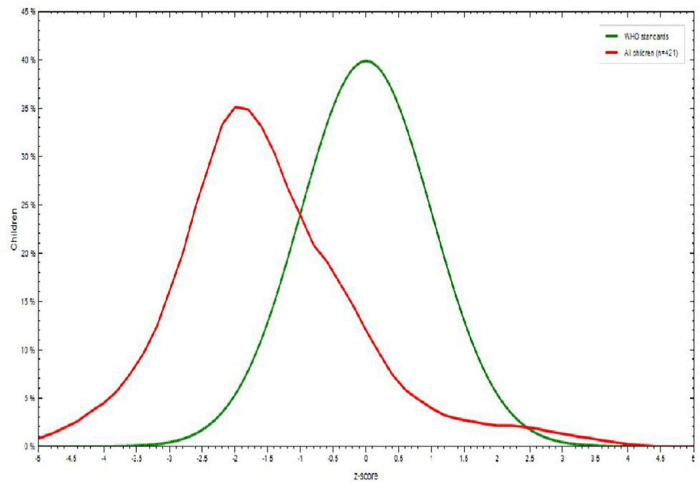
Height-for-age *Z* score among children aged 6–23 months in Dessie town, Northeastern Ethiopia, 2021.

Among children aged 6-23 months, 153 (36.8%) stunted. There was little difference between boys (93, 60.8%) and girls (60, 39.2%). There was also a noticeable difference between children with a mother in family planning use 142 (92.8%) and not used 11 (7.2%). Children 12-17 months of age showed the highest percentage of stunting 52 (33.12%) (Figure 1).

### Underweight/Weight for Age/

**FIGURE 2:**
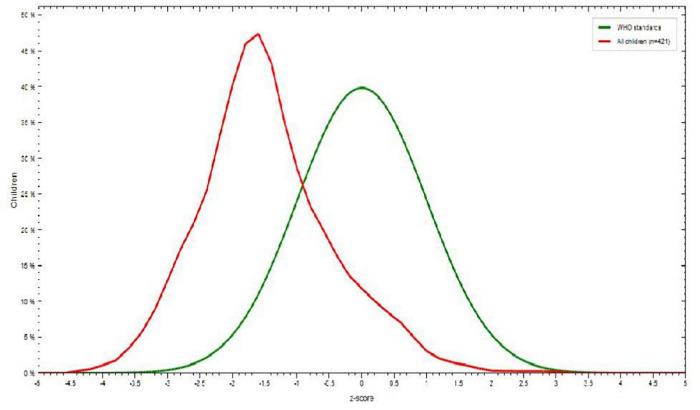
Weight-for-age *Z* score among children aged 6–23 months in Dessie town, Northeastern Ethiopia, 2021.

Among children 6-23 months of age, 115 (27.6%) were underweight. Children 6-8 months of age showed the highest percentage of underweight 41 (35.6%), children 12-17 months of age were less likely to be underweight, and males 76 (66.1%) were more underweight than females 39 (33.9%) (Figure 2).

### Wasting/Weight-for-Height/

**FIGURE 3:**
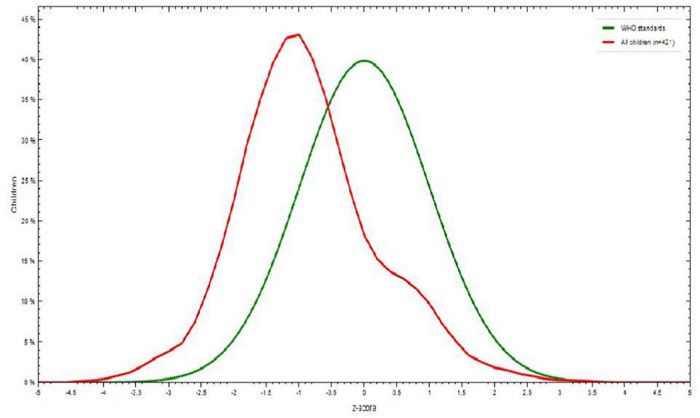
Weight-for-height *Z* score among children aged 6–23 months in Dessie town, Northeastern Ethiopia, 2021.

Among children aged 6-23 months, 48 (11.5%) were wasted. There was little difference between boys (28, 58.3%) and girls (20, 41.7%). Children aged 12-17 months were wasted 19 (39.5%) compared to children aged 6-8 months (Figure 3).

### Factors Associated With Stunting

The results of multivariable logistic regression analysis showed that sex of the child, maternal family planning and maternal or caregiver handwashing practices were statistically associated with stunting at a *p* value <0.05. As shown in Table 9, among the variables entered into the multivariable logistic regression analysis, male children were almost 1.55 times more likely to be stunted than female children (AOR = 1.55, 95% CI: 1.02, 2.34). Children whose mothers had used family planning services had a 0.38 times lower chance of developing stunting than children whose mothers had not used family planning services (AOR = 0.38, 95% CI: 0.19, 0.77).

**TABLE 7:** Factors associated with stunting in Dessie town, Northeastern Ethiopia, 2021.

Variable	Stunting		COR (95%CI)	AOR (95%CI)	*p* value
	Yes	No			
Sex of the child Male Female	93(22.4%) 60(14.4%)	131(31.5%) 132(31.7%)	1.56(1.04,2.34) 1	1.55(1.02, 2.34) 1	0.038*
Paternal occupation Civil servant Self-employer Daily worker	90(21.6%) 44(10.6%) 19(4.6%)	123(29.6%) 98(23.5%) 42(10.1%)	1 1.63(1.04,2.55) 1.62(0.88,2.97)	1 1.66(1.05, 2.62) 1.32(0.70, 2.49)	0.29 0.384
Family planning use No Yes	11(2.6%) 142(34.1%)	43(10.3%) 220(53%)	1 0.40(0.19,0.78)	1 0.38(0.19,0.77)	0.01*
Handwashing practice If soap/ash used Neither soap/ash used	144(34.6%) 9(2.2%)	230(55.3%) 33(7.9%)	1 2.30(1.07,4.94)	1 2.32(1.05, 5.11)	0.037*

“*” = Meaning significantly associated variables with undernutrition, 1 = Reference.

**TABLE 8:** Factors associated with underweight in Dessie town, Northeastern Ethiopia, 2021.

Variable	Underweight		COR (95%CI)	AOR (95%CI)	*p* value
	Yes	No			
Child age 6-8 months 9-11 months 12-17 months	41(9.8%) 39(9.4%) 35(8.4%)	48(11.5%) 61(14.7%) 192(46.2%)	1 1.34(0.75, 2.38) 4.69(2.70, 8.13)	1 1.36(0.76, 2.45) 4.62(2.65, 8.06)	0.303 0.01*
Sex of the child Female Male	39(9.4%) 76(18.3%)	153(36.8%) 148(35.5%)	1 2.02(1.29, 3.15)	1 1.93(1.21, 3.07)	0.006*
Child breastfeeding Yes No	82(19.7%) 33(7.9%)	169(40.7%) 132(31.7%)	1 1.94(1.22, 3.09)	1.48(0.89, 2.46)	0.126
Complete immunization No Yes	47(11.3%) 68(16.3%)	71(17.1%) 230(55.3%)	1 2.25(1.42,3.54)	1 0.98(0.47,2.04)	0.95

“*” = Meaning significantly associated variables with undernutrition, 1 = Reference.

**TABLE 9:** Factors associated with wasting in Dessie town, Northeastern Ethiopia, 2021.

Variable	Wasting		COR (95%CI)	AOR (95%CI)	*p* value
	Yes	No			
Child age 6-8 months 9-11 months 12-17 months	14(3.4%) 15(3.6%) 19(4.6%)	75(18%) 85(20.4%) 208(50%)	1 1.06(0.48, 2.34) 2.04(0.98, 4.28)	1 1.12 (0.50, 2.46) 2.25(1.06, 4.78)	0.81 0.035*
Child ARTI Yes No	6(1.4%) 42(10.1%)	64(15.4%) 304(73.1%)	1 0.68(0.28, 1.66)	1 0.73(0.29,1.84)	0.503
Treatment of drinking water Not treated Treated	42(10% 6(1.4%)	356(85.6%) 12(3%)	1 0.24(0.09,0.66)	1 0.21(0.07,0.59)	0.03*
Handwashing practice Neither soap/ash used Soap/ash used	8(2.0%) 40(9.6%)	34(8.2%) 334(80.2%)	1 1.96(0.85, 4.54)	1.48(0.59, 3.68)	0.39

“*” = Meaning significantly associated variables with undernutrition, 1 = Reference.

The odds of being stunted from children’s families who had not washed their hands with soap or ash were 2.32 times higher when compared to children’s families who washed their hands with soap or ash (AOR = 2.3295% CI: 1.05, 5.11) (Table 7).

### Factors Associated With Underweight

Multivariable logistic regression analysis for underweight revealed that the age of the child and sex of the child were statistically associated with an underweight *P* value <0.05 level of significance.

The odds of underweight among children aged 12-17 months were approximately 4.62 times higher than those among children aged 6-8 months (AOR = 4.62, 95% CI: 2.65, 8.06).

The probability of underweight in male children was found to be 1.93 times higher than that of female children (AOR = 1.93, 95% CI: 1.21, 3.07) (Table 8).

### Factors Associated With Wasting

Multivariate logistic regression analysis revealed that child age difference and household handwashing practice were statistically associated with wasting with a *P* value <0.05 level of significance.

The odds of wasting from children aged 12-17 months were approximately 2.25 times more likely to be affected by wasting than children aged 6-8 months (AOR = 2.25, 95% CI: 1.06, 4.78).

The probability of wasting from children’s families that had not taken treatment on drinking water was approximately 0.21 times affected by wasting compared with children’s families taking treatment on drinking water (AOR = 0.21, 95% CI: 0.07, 0.59) (Table 9).

#### Discussion

The findings of this study showed that the prevalence of stunting was 36.8%, which is in line with other survey studies performed in Rah Kin state of Myanmar in 2009-2010 (7), the Bahari division, Kilifi County and in Mbeere district Kenya (12, 13), which were reported to be 37.4, 39.7, and 39%, respectively. This similarity might be due to the child feeding habits and socioeconomic characteristics of the population. In contrast, it is different from the EDHS results of the Amhara Region (11), Somali Region (14), Afambo District and Agro pastorals in northern Ethiopia (15), where the prevalence of stunting was reported to be 22.9 and 32.2%, respectively. This might be due to differences in socioeconomic characteristics, demographics and culture in child care and feeding. In this study, sex was significantly associated with the development of stunting. Males are nearly two times more likely to be stunted than females. This finding is similar to other studies performed in Labella Town, Ethiopia (15). The cause of the discrepancy in sex is not well established in other studies. However, it is believed that boys are more influenced by environmental stress than girls (16). In this study, handwashing practice was also significantly associated with stunting. The odds of stunting among children whose families did not wash their hands with soap/ash were 2.32 times more likely to have stunting compared with families who wash their hands with soap/ash. This finding is similar to those of other studies performed in eastern India, Jharkhand and Modish (17). The possible reason may be due to the higher risk of infection during breast/formula feeding and caring in women who do not wash their hands. Family planning was also significantly associated with stunting. This finding is in line with other studies performed in eastern India, Jharkhand and Modish (17). This might be because if the mother does not use contraceptives, the inter-pregnancy interval will be short. As a result, children will not receive adequate care and feeding, which results in stunting.

This study also revealed that the prevalence of underweight was 27.6%, which is similar to the EDHS results of the Amhara and Somali regions (11), which reported prevalence rates of 28.4% and 28.7%, respectively. In contrast, it is different from other studies performed in Afar, Benishangul Gumuz (7) and Bahari Division, Kilifi Country (12), where the prevalence of underweight was reported to be 36.2, 34.3, and 39.7%, respectively. This difference might be due to differences in sample size, wide area coverage and cultural variation in child care and feeding. Based on this finding, the age of the child was significantly associated with the development of underweight. Children aged 12-17 months were 4.62 times more likely to be underweight than those less than 6-8 months of age. This finding is similar to studies performed in Mbeere South District Kenya (13). This might be linked to an inadequate frequency of breast feeding (<12 times per day) and the requirement of more supplemental foods. Sex was another variable associated with underweight. Males were nearly 0.53 times more likely to be underweight than females. This finding is similar to other findings obtained in Tigray in Saesie Tsaeda-Emba district (18). This might be due to feeding habits and sociodemographic and cultural similarities in child care and feeding. The findings of this study showed that the prevalence of wasting was 11.5%, which is in accord with the regional prevalence of Benishangul-Gumuz (11.5%) and Tigray (11.1%) reported by EDHS 2016 (9). However, the prevalence in the study area was lower than that in studies performed in the Amhara region in East Belesa district, North eastern Ethiopia (14), Somali, Ethiopia (14), Kemba Woreda (19) and Bahari Division, Kilifi County (12), with wasting prevalence rates of 16, 17.5, 21, and 41%, respectively. However, the prevalence of wasting in the study area is higher than the 9.8% from Lalibela, North Wollo Ethiopia (16), 8.9% from the Amhara region (11) and 10.8% from the Myanmar Multiple Indicator Cluster Survey (MICS) (20). These discrepancies may be due to variations in the data collection period, cultural differences in childcare and feeding and wide area coverage.

In this study, treating drinking water was significantly associated with the development of wasting. Families who had treated drinking water were 79% less likely to have developed wasting than families who had untreated drinking water. This finding is in line with studies performed in Guto Gida District, Oromia Regional State, Ethiopia (21). The possible reason is that treating water will prevent infection and secondary malnutrition. In this study, child age was significantly associated with the development of wasting. Children aged 12-17 months were 2.25 times more likely to have wasting than those less than 6-8 months of age. This result is similar to those of studies performed in Guto Gida District, Oromia Regional State, Ethiopia (21). This might be due to similarities in socioeconomic, sociodemographic and access to modern health facilities. The possible reason might be linked to an inadequate frequency of breast feeding (<12 times per day) and the requirement of more supplemental foods.

## Conclusion

In this study, the prevalence of undernutrition among children aged 6-23 months was higher for stunting (36.8%), underweight (27.6%) and wasting (11.5%) compared to WHO classification. Sexes of the child, maternal family planning and handwashing practice were significantly associated with stunting. The likelihood of developing wasting was associated with child age and families taking action on drinking water, and child sex and age were significantly associated with underweight.

## Data Availability Statement

The raw data supporting the conclusions of this article will be made available by the authors, without undue reservation.

## Ethics Statement

The studies involving human participants were reviewed and approved by Wollo University College of medicine and health science ethical review committee. Written informed consent to participate in this study was provided by the participants’ legal guardian/next of kin.

## Author Contributions

TS contributed to conception and design of the study. TS and MWS organized the database. TS, MWS, YZ, WY, and LW performed the statistical analysis. MWS wrote the first draft of the manuscript. TS, MWS, YZ, WY, and LW wrote sections of the manuscript. All authors contributed to manuscript revision, read, and approved the submitted version.

## Conflict of Interest

The authors declare that the research was conducted in the absence of any commercial or financial relationships that could be construed as a potential conflict of interest.

## Publisher’s Note

All claims expressed in this article are solely those of the authors and do not necessarily represent those of their affiliated organizations, or those of the publisher, the editors and the reviewers. Any product that may be evaluated in this article, or claim that may be made by its manufacturer, is not guaranteed or endorsed by the publisher.

## Abbreviations

ANC: Antenatal Care
ARTI: Acute Respiratory Tract Infection
DDS: Dietary Diversity Score
DRC: Democratic Republic of Congo
EDHS: Ethiopia Demographic Health Survey
EPI: Expanded Program on Immunization
FP: Family Planning
FANTA: Food and Nutrition Technical Assistant
FAO: Food and Agricultural Organization
HFIAS: Household Food Insecurity Access Scale
IFAD: International Fund for Agricultural Development
IYCF: Infant and Young Child Feeding
MICS: Multiple Indicator Cluster Survey
NCHS: National Center for Health Statistics
PI: Principal Investigator
PNC: Postnatal Care
SBCC: Social and Behavioral Change Communication
SECA TB: Southern Early Childhood Association Tuberculosis.
